# Potential regulatory role of PGC-1α within the skeletal muscle during metabolic adaptations in response to high-fat diet feeding in animal models

**DOI:** 10.1007/s00424-023-02890-0

**Published:** 2023-12-04

**Authors:** Sinenhlanhla X. H. Mthembu, Sithandiwe E. Mazibuko-Mbeje, Khanyisani Ziqubu, Ndivhuwo Muvhulawa, Fabio Marcheggiani, Ilenia Cirilli, Bongani B. Nkambule, Christo J. F. Muller, Albertus K. Basson, Luca Tiano, Phiwayinkosi V. Dludla

**Affiliations:** 1 Biomedical Research and Innovation Platform, South African Medical Research Council, Tygerberg, Cape Town, 7505 South Africa; 2 Department of Biochemistry, North-West University, Mafikeng Campus, Mmabatho, 2735 South Africa; 3 Department of Life and Environmental Sciences, Polytechnic University of Marche, 60131 Ancona, Italy; 4 Department of Clinical Sciences, Section of Biochemistry, Polytechnic University of Marche, 60131 Ancona, Italy; 5 School of Laboratory Medicine and Medical Sciences, University of KwaZulu-Natal, Durban, 4000 South Africa; 6 Centre for Cardiometabolic Research Africa (CARMA), Division of Medical Physiology, Stellenbosch University, Tygerberg, Cape Town, 7505 South Africa; 7 Department of Biochemistry and Microbiology, University of Zululand, KwaDlangezwa, Empangeni, 3886 South Africa; 8 Cochrane South Africa, South African Medical Research Council, Tygerberg, 7505 South Africa

**Keywords:** Obesity, High-fat diet, Mitochondrial function, Insulin resistance, Skeletal muscle, PGC-1α

## Abstract

High-fat diet (HFD) feeding in rodents has become an essential tool to critically analyze and study the pathological effects of obesity, including mitochondrial dysfunction and insulin resistance. Peroxisome proliferator–activated receptor γ coactivator-1α (PGC-1α) regulates cellular energy metabolism to influence insulin sensitivity, beyond its active role in stimulating mitochondrial biogenesis to facilitate skeletal muscle adaptations in response to HFD feeding. Here, some of the major electronic databases like PubMed, Embase, and Web of Science were accessed to update and critically discuss information on the potential role of PGC-1α during metabolic adaptations within the skeletal muscle in response to HFD feeding in rodents. In fact, available evidence suggests that partial exposure to HFD feeding (potentially during the early stages of disease development) is associated with impaired metabolic adaptations within the skeletal muscle, including mitochondrial dysfunction and reduced insulin sensitivity. In terms of implicated molecular mechanisms, these negative effects are partially associated with reduced activity of PGC-1α, together with the phosphorylation of protein kinase B and altered expression of genes involving nuclear respiratory factor 1 and mitochondrial transcription factor A within the skeletal muscle. Notably, metabolic abnormalities observed with chronic exposure to HFD (likely during the late stages of disease development) may potentially occur independently of PGC-1α regulation within the muscle of rodents. Summarized evidence suggests the causal relationship between PGC-1α regulation and effective modulations of mitochondrial biogenesis and metabolic flexibility during the different stages of disease development. It further indicates that prominent interventions like caloric restriction and physical exercise may affect PGC-1α regulation during effective modulation of metabolic processes.

## Introduction

Pathophysiological mechanisms elucidating the development of insulin resistance have been increasingly explored for their relevant in curbing metabolic diseases [45, 49, 70]. With accumulative evidence highlighting the significant role of high-fat diet (HFD) feeding in driving the initiation and progression of both insulin resistance and mitochondrial dysfunction [45, 49, 70]. Firstly, it has been argued that HFD feeding can interfere with mitochondrial oxidative capacity, which is mainly modulated through reduced expression of peroxisome proliferator–activated receptor γ coactivator-1α (PGC-1α) in rodents [74]. Secondly, others have indicated that HFD can instigate muscle insulin resistance by promoting mitochondrial biogenesis [21]. This was shown to be facilitated through activation of peroxisome proliferator–activated receptor (PPAR)δ, which mediates the posttranscriptional increase of PGC-1α [21]. Effective modulation of PGC-1α, together with related sirtuin 1 (SIRT1) and 5′ AMP-activated protein kinase (AMPK) signaling mechanism, remains crucial to improve cellular metabolism and to promote skeletal muscle recovery [65, 69, 76]. Likewise, interaction of PGC-1α with transcriptional factors (PPARs) is required for effective control essential metabolic processes, involving cellular energy production, thermogenic activities, and lipid metabolism [14, 16, 42].

Obviously, depending on the duration of feeding, different research groups have explored different perspectives in terms of how HFD contributes to the development of metabolic anomalies and obesity, including insulin resistance, intramuscular lipid droplet accumulation, and mitochondrial function [4, 5, 20]. For example, it has been observed that enhanced muscle mitochondrial oxidative capacity could occur independent of PGC-1α regulation in response to HFD feeding, while prominent interventions like physical activity could promote metabolic health by effectively regulating PPAR proteins in rodents [21, 27, 35, 47]. We have previously reviewed evidence on the implications of lipid overload and its potential contribution to the development of skeletal muscle insulin resistance and pathological changes in mitochondrial oxidative capacity [60], without focusing on the molecular mechanisms that could be involved in this process. Therefore, because of its significant role in controlling energy metabolism and involvement in insulin signaling [23, 73, 86], it remains important to establish how HFD affects skeletal muscle function in preclinical models of obesity. Special attention falls on the causal relationship between regulation of PGC-1α in connection with the development of mitochondrial dysfunction and insulin resistance within the skeletal muscle.

This review also uniquely covers information related to the influence of prominent interventions like caloric restriction and physical exercise on skeletal muscle in response to HFD, especially elucidating the connection between PGC-1α regulation and improved metabolic function. To identify relevant studies discussed in the review, a systematic search was conducted by focusing on electronic databases such as PubMed, Embase, and Web of Science using medical subject heading (MeSH) terms such “insulin resistance,” “PGC-1α,” “mitochondria,” “skeletal muscle,” and “high fat-diet.” A similar and detailed method for study inclusion has already been explained in other publications [87].

## A general overview of PGC-1α and its potential role in regulating skeletal muscle function

PGC-1α was initially discovered as a cold-inducible transcription coactivator of adaptive thermogenesis [42]. It is now widely known as a member of the family of transcription coactivators that known to be instrumental in the regulation of cellular energy metabolism and mitochondrial biogenesis [82]. The regulation of PGC-1α is controlled by several signaling cascades, proteins, and several transcription factors. For example, in skeletal muscle, PGC-1α interacts with numerous transcription factors involved in mitochondrial biogenesis such as mitochondrial transcription factors (TFAM), nuclear respiratory factors (NRFs), estrogen-related receptors (ERRs), and PPARs ([14, 16, 42]; [7, 64]). This extends to its regulation of cAMP response element-binding protein (CREB) and free fatty acid (FFA) oxidation in skeletal muscle in response to increased physical exercise [3, 36, 46, 82]. Essentially, PGC-1α in combination with these transcriptional factors plays a huge role in mitochondrial proliferation and cell respiration and regulation of lipid metabolism in many tissues [3, 26, 36, 46, 82].

During the physical exercise, calcium signaling cascades in combination with CREB have been shown to activate this transcriptional factor within the skeletal muscle in preclinical models [26, 37]. Some studies reported that overexpression of PGC-1α promotes glucose uptake, which directly improves insulin sensitivity, through enhanced expression of glucose transporter 4 (GLUT4) in cultured muscle cells [52, 66]. Alternatively, PGC-1α can also promote FFA oxidation while blocking glycolysis and utilization of glucose within skeletal muscle [56]. Besides regulating mitochondrial function in muscle, PGC-1α is pivotal for modulating other skeletal muscle processes, such as regulating protein degradation, autophagy, satellite cell function, endoplasmic reticular stress, and inflammatory responses [17, 30, 71].

Over the past years, research revealed that PGC-1α expression is dysregulated in key metabolic tissues of animals and humans with insulin resistance and type 2 diabetes (T2D) [68, 82]. In the skeletal muscle of humans with T2D and prediabetic individuals, PGC-1α expression and its co-transcription activity were reduced, in parallel with the suppressed mitochondrial biogenesis and mitochondrial oxidative capacity [55, 68, 82]. In vitro evidence has shown that the expression of PGC-1α is reduced in skeletal muscle cells with palmitate-induced insulin resistance and mitochondrial dysfunction [80]. The similar effect was also observed in mice and rats exposed to HFD feeding [72, 80]. Others have shown that skeletal muscle-specific PGC-1α knockout reduces muscle endurance capacity and damage to muscle fibers following treadmill running [22]. Another hypothesis prevails that PGC-1α triggers the expression of genes involved in lipid transport or storage as well as their utilization, thus potentially reflecting its important physiological role in metabolic adaptations to physical exercise [37].

Increasing studies have certainly indicated that focusing in the PGC-1α for its potential role during the development of insulin resistance, mitochondrial dysfunction, and therefore of T2D metabolism [14, 16, 42, 46, 65]. Even though most studies acknowledge the important role of PGC-1α during the regulation of mitochondrial substrate utilization and insulin resistance within the skeletal muscle [33, 52, 66], much remains to be discovered concerning disease development and progression thereof, especially in response to HFD-feeding.

## Potential regulation of PGC-1α within the skeletal muscle in response to partial exposure to HFD feeding

It has become increasingly clear that besides determining the composition of a diet [75], the duration of feeding such diet remains important to induce the desired pathological effect(s). Others have even argued that implementation of standard protocols that better mimic effects on fetal growth seen in obese humans will improve clinical relevance of results [15]. Anyway, in most rodent-based preclinical models, a HFD contains 60% fat [54]. However, it is also true that animals can present with varied pathological features, depending on the duration to which animals they are exposed to HFD. Using C57BL/6 mice, Lee and colleagues showed that as early as 2 weeks of HFD feeding was enough to negatively affect mitochondrial function, including decreasing citrate synthase activity, mitochondrial respiration, and mitochondrial DNA within the skeletal muscle [39]. Interestingly, these results were consistent with reduced skeletal muscle insulin sensitivity in these mice, while no effect was observed in the liver. Verifying the already discussed hypothesis [2, 60], skeletal muscle insulin resistance and mitochondrial dysfunction are strongly interconnected and may develop rather early in mice, even before any other obvious pathological changes.

**Fig. 1:**
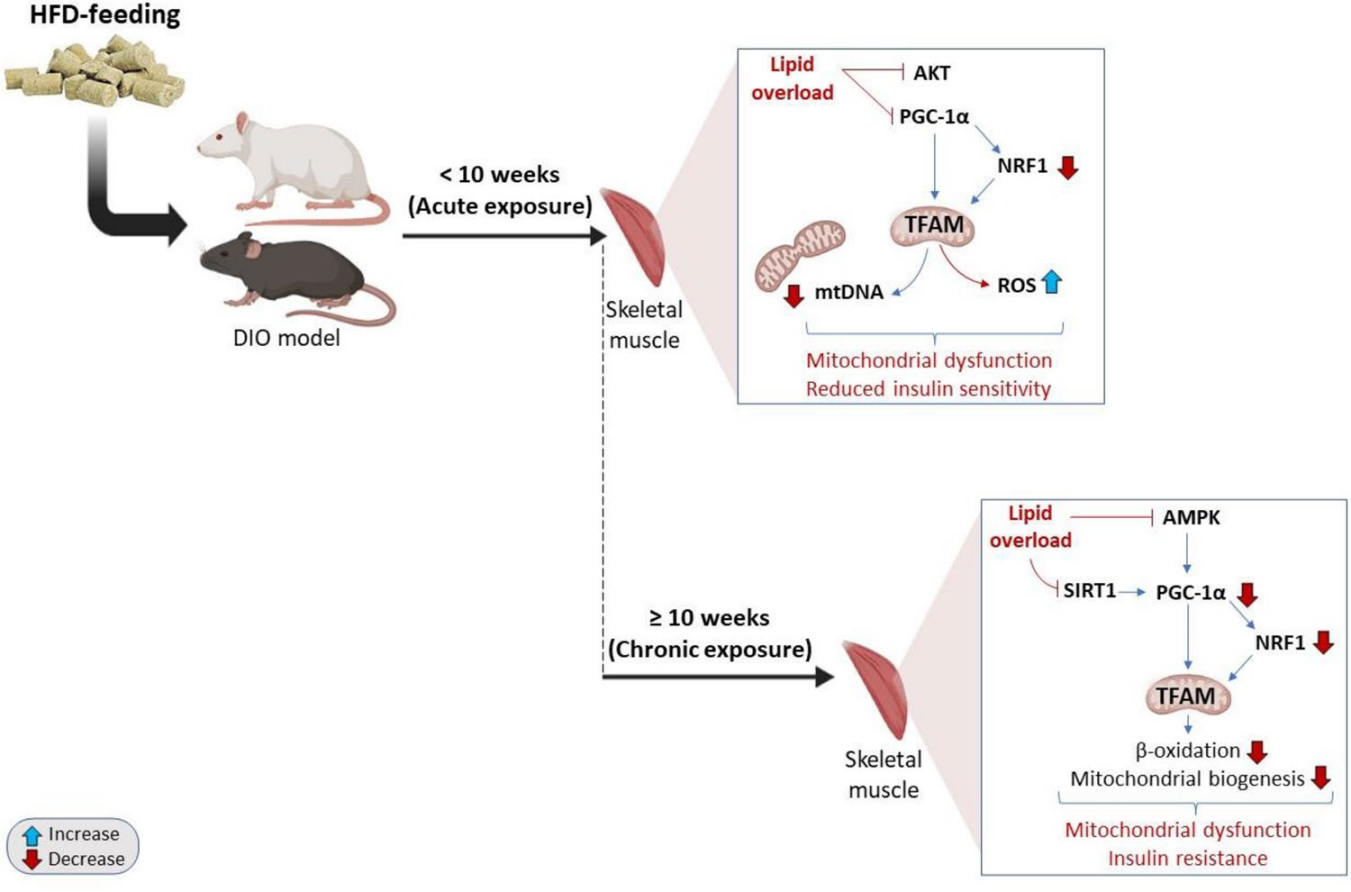
An overview of mechanisms depicting the detrimental effects of high-fat diet (HFD) on skeletal muscle function in preclinical models of obesity. Briefly, HFD feeding (lipid overload) can hinder the efficiency of the mitochondria by interfering with PGC-1α activity, driving reactive oxygen species (ROS) production and oxidative stress within the skeletal muscle. This process is further associated with intracellular antioxidant responses (through NRF1) and altered energy metabolism, through altered AMP-activated protein kinase (AMPK) and Sirt1 (member of the sirtuin family), leading to insulin resistance in response to HFD feeding in animals

Table 1 gives an overview of preclinical studies reporting on the effects of acute or short-term (< 10 weeks) HFD on potential regulation of PGC-1α, comparing its pathological implication during the development of mitochondrial dysfunction within the skeletal muscle (Fig. 1). Interestingly, a study by Li and colleagues [41] reported that alternating HFD for 4 weeks could enhance mitochondrial enzyme activities and protein content in rat skeletal muscle, although there were no significant changes with muscle glycogen concentration or glucose transport. However, most of the summarized evidence suggest that an average time of 3–4 weeks of HFD feeding in mice is sufficient to impair skeletal muscle mitochondrial function, and this is mainly through an alteration in cellular respiratory processes [29, 48, 53]. Certainly, the predominant molecular mechanisms involved during this process mainly involve reduced expression (both protein and mRNA) of PGC-1α, which may concomitantly suppress other mitochondrial function regulating transcriptional factors like NRF1 and TFAM that are necessary for an efficient cellular respiration process. Interestingly, such detrimental effects within skeletal muscle are observed even if mice are exposed to HFD feeding for 8 weeks [25, 80], with reduced insulin sensitivity and increased ROS impairing mitochondrial function or respiratory process within the skeletal muscle of these mice [25, 29, 48, 53, 80]. Blocking the phosphorylation of protein kinase B (Akt), which is normally required for modulating metabolic effects of insulin within the skeletal muscle [28], appears to be the main mechanism causing reduced insulin sensitivity or driving the development of insulin resistance [48], further suggesting that alterations in mitochondrial respiration and reduced insulin sensitivity drive pathological abnormalities of HFD feeding.

**Table 1:** Evidence on the potential regulation of peroxisome proliferator–activated receptor gamma coactivator 1-alpha (PGC-1α) in response to partial exposure to high-fat diet (HFD) feeding within the skeletal muscle in preclinical models

Author, year	Experimental model and duration of HFD feeding	Experimental outcome
Short-term exposure to HFD feeding (< 10 weeks)		
Hong et al., 2016 [25]	Male C57BL/6J mice fed a HFD for 8 weeks	Caused insulin resistance (IR) and suppressed mitochondrial oxidative phosphorylation, fatty acid oxidation enzymes and uncoupling proteins, including protein expression of UCP2/3 and PGC-1α
Li et al., 2016 [41]	Male Wistar rats fed a HFD for 4 weeks	Increased mitochondrial enzyme activities and protein content of PGC-1α
Ju et al., 2017 [29]	Female C57BL/6J mice were subjected to under-nutrition and male offspring assigned to HFD for 21 days (3 weeks)	Caused glucose intolerance, IR, and suppressed the mRNA expression of mitochondrial DNA (mtDNA), PGC-1α, NRF1, and TFAM
Martins et al., 2018 [48]	Male C57BL/6 mice were fed HFD supplemented with fish oil for 4 weeks	Inhibited protein kinase B (Akt) phosphorylation, reduced oxygen consumption, tricarboxylic acid cycle intermediate contents (citrate, α-ketoglutarate, malate, and oxaloacetate), and reduced PGC1-α transcription.
Miotto et al., 2018 [53]	Male C57Bl6J mice were fed HFD for 4 weeks	Caused IR, increased protein expression of PGC-1α, and reduced the sensitivity of ADP, leading to increased mitochondrial hydrogen peroxide (H_2_O_2_) emission
Xu et al., 2019 [80]	Male C57BL6/J mice fed HFD for 8 weeks	Caused glucose intolerance while suppressing the protein expression of PGC-1α and TFAM leading to muscle degeneration

## Potential regulation of PGC-1α regulation within the skeletal muscle in response to chronic exposure to HFD feeding

Given that many facets of the metabolic disease are still not completely understood, animal models have undoubtedly become fundamental in providing a platform to uncover pathological mechanisms that may be involved during the early development or progression of this condition [19]. Acute or short-term HFD feeding (< 10 weeks) is already accredited with the development of many pathologies, including impairing mitochondrial respiration processes and initiating insulin resistance, which occurs in part through reducing the expression of PGC-1α within the skeletal muscle (Table 1). Even more essential to understand are the consequences of chronic or long-term HFD in rodents, especially since no single animal model comprehensibly mimics all pathophysiological features and natural history of the metabolic syndrome. In fact, accumulative research supports the notion that HFD feeding aggravates insulin resistance, alters eating behavior, exacerbates dyslipidemia, and can even lead to skeletal muscle wasting in rodents [1, 4, 57]. Thus, it remains essential to decipher how HFD feeding modulates PGC-1α expression or activity in relation to mitochondrial function or even insulin signaling within the skeletal muscle.

Table 2 gives an overview of preclinical studies reporting on the effects of chronic or long-term (≥ 10 weeks) HFD on the potential modulation of PGC-1α within the skeletal muscle, to decipher implicated pathological mechanisms that might contribute to skeletal muscle dysfunction (Fig. 1). Starting from 10 weeks, it is reported that HFD feeding significantly reduced PGC-1α gene (mRNA) expression within the skeletal muscle of C57BL/6J mice, and this was linked with altered mitochondrial adaptation, impaired β-oxidation, and development of insulin resistant phenotype [24]. However, as from 12 weeks, HFD feeding did not affect or rather enhanced the expression (protein/mRNA) of PGC-1α within the skeletal muscle of Sprague–Dawley rats [81, 83]. These effects were also linked with reduced protein expression of AMPK, SIRT3, and mitochondrial biogenesis, which are important regulators of energy metabolism and oxidative phosphorylation [44, 84]. What certainly became clear is that prolonged HFD feeding, from 16–18 weeks, certainly impedes the efficiency or activity of the mitochondrial chain function and leads to the development of insulin resistance within the skeletal muscle of mice [72, 79]. However, these pathological changes are not linked with PGC-1α expression or are rather associated with its enhanced activity within the skeletal muscle of these mice, further indicating the importance of this transcriptional factor in modulating an adaptive response, stimulating mitochondrial biogenesis, and favoring the recovery of skeletal muscle in conditions of stress, as reviewed elsewhere [31, 40].

**Table 2:** Evidence on potential regulation of peroxisome proliferator–activated receptor gamma coactivator 1-alpha (PGC-1α) in response to chronic exposure to high-fat diet (HFD) feeding within the skeletal muscle in preclinical models

Author, year	Experimental model and duration of HFD feeding	Experimental outcome
Long-term exposure to HFD feeding (≥ 10 weeks)		
Henagan et al., 2015 [24]	Male C57BL/6J mice fed a HFD for 10 weeks	Reduced PGC-1α gene (mRNA) expression and impaired β-oxidation which favored insulin resistant (IR) phenotype
Zhang et al., 2015 [83]	Male Sprague–Dawley rats fed HFD for 12 weeks	Caused IR and reduced protein expression of sirtuin 1/3 (SIRT1/3). However, overexpression of SIRT1 ameliorated skeletal muscle IR and increased mRNA expression of 5′ AMP-activated protein kinase (AMPK), PGC-1α, SIRT3, and mitochondrial biogenesis
Pileggi et al., 2016 [67]	Female Sprague-Dawley rats exposed to maternal HFD for 10 days. At weaning (day 21), male siblings from each litter were fed standard chow diet for the remainder of the study (day 150)	HFD offspring exhibited reduced expression of mitochondrial transcription factors nuclear respiratory factor (NRF)-1 and mitochondrial transcription factor A (mtTFA), however PGC-1α and NRF2 gene (mRNA) expression was not affected
Wang et al., 2016 [79]	Male C57BL/6 mice fed a HFD for 16 weeks	Enhanced mitochondrial O-GlcNAcylation that was complemented by decreased protein expression levels of PGC-1α and mitochondrial density
Liu et al., 2017 [43]	Male Sprague-Dawley rats fed HFD supplemented with 1.5% leucine for 24 and 32 weeks	Caused mitochondrial dysfunction in skeletal muscle and early stage of IR after 24 weeks, including enhancing the expression of genes (mRNA expression) involved in mitochondrial biogenesis like TFAM, NRF1, PGC-1α and SIRT1
Shen et al., 2019 [72]	Male C57BL/6J mice fed HFD for 18 weeks	Induced adiposity and IR through promoting lipid accumulation, while suppressing protein expression levels of PGC-1α, cyclooxygenase-2 (COX2), cluster of differentiation 36 (CD36), and UCP3
Lee et al., 2021 [39]	Male C57BL/6 mice fed HFD for 2 weeks (induce acute IR) and/or for 24 weeks (induce chronic IR)	HFD for short term decreased citrate synthase activity, complex I and II in muscle tissue but opposite results were observed with HFD for long term which increased complex I, IV and decreased complex II. Interestingly, both diets did not affect protein expression of PGC-1α/β and peroxisome proliferator-activated receptor (PP AR) δ
Yeo et al., 2022[81]	Female Sprague-Dawley rats fed HFD for 12 weeks	Decreased the activity of citrate synthase and the content of cytochrome C Oxidase Subunit 4 (COX4). However, PGC-1α protein expression was not affected by HFD

Furthermore, it was even more clear that HFD feeding exceeding 24 weeks does not affect the protein or mRNA expression of PGC-1α or its associated peroxisome proliferator–activated receptor (PPAR)δ in mice [39]. This may indicate that prolonged HFD feeding favors irreversible pathological modifications that severely affect skeletal muscle function and can even lead to muscle wasting [1]. Apparently, adding the essential amino acid leucine (at 1.5%) as part of HFD for at least 24 weeks led to an incompletely oxidized lipid species that contributed to mitochondrial dysfunction in skeletal muscle of HFD-fed Sprague-Dawley rats in the early stage of insulin resistance [43]. Interestingly, these effects can be reversed, and skeletal muscle mitochondrial function improved in offspring of Sprague-Dawley rats that were initially maintained in HFD for 10 days prior to mating and throughout pregnancy and lactation [67].

## Therapeutic interventions that affect skeletal muscle function in response to HFD feeding

**Fig. 2:**
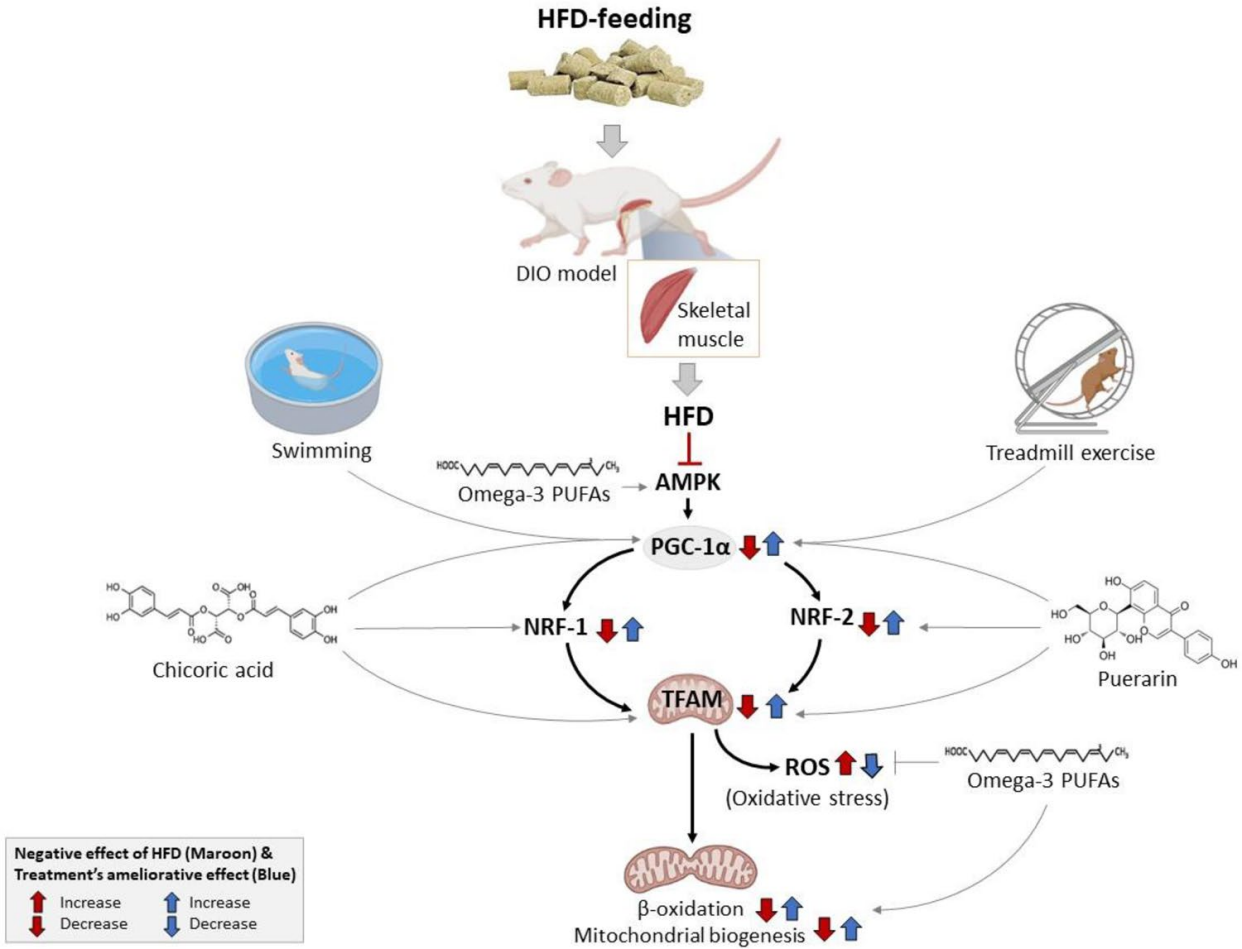
An overview of various intervention strategies to improve mitochondrial function in skeletal muscle after high-fat diet (HFD) feeding in diet-induced obesity (DIO) model. Briefly, combining caloric restriction and endurance exercise can improve mitochondrial biogenesis within the skeletal muscles of Wistar rats fed with HFD. In addition to potentially affecting PGC-1α expression, these effects were consistent with the amelioration of insulin resistance and reduction in toxic levels of oxidative stress, including improved mitochondrial dynamics and skeletal muscle function. More studies are still required to confirm the potential role of bioactive substances like omega-3 rich foods, chicoric acid, and puerarin that targets PGC-1α to ameliorate HFD-induced skeletal muscle pathologies

Table 3 gives an overview of preclinical studies on the potential effects of some interventions in modulating mitochondrial function, while also affecting targeting PGC-1α within the skeletal muscle in response to HFD feeding (Fig. 2). Starting with diet modification, protein restriction for 6 weeks before HFD feeding was effective in reducing body weight gain and fat accumulation, and this outcome was consistent with the activation and improvement of skeletal muscle energy expenditure in C57BL/6 mice [10]. Alternatively, giving Wistar rats a diet containing omega (ω)-3 polyunsaturated fatty acids (PUFAs) for 6 weeks could promote lipid oxidation and decrease energy efficiency in subsarcolemmal mitochondria, while activating AMPK and reducing both endoplasmic reticulum and oxidative stress in these animals [12]. Importantly, these effects were consistent with enhanced mitochondrial respiration and increased PGC-1α expression and mitochondrial biogenesis within the skeletal muscle in these Wistar rats fed a diet rich in PUFAs [12].

**Table 3:** Therapeutic interventions that may potentially affect peroxisome proliferator–activated receptor gamma coactivator 1-alpha (PGC-1α) within the skeletal muscle in response to high-fat diet (HFD) feeding

Author, year	Experimental model and duration of HFD feeding	Experimental outcome
Cavaliere et al., 2016 [12]	Male Wistar rats fed a diet containing omega (ω)-3 polyunsaturated fatty acids (PUFAs) for 6 weeks	PUFAs promoted lipid oxidation and reduced energy efficiency in subsarcolemmal mitochondria, while activating AMPK and decreasing both endoplasmic reticulum and oxidative stress. Further, enhanced PGC-1α mRNA expression
de Queiroz et al., 2017 [6]	Male Wistar rats fed a high sugar diet and exposed to 60 min of regular physical activity by swimming (without workload) for 4 and 8 weeks	High sugar diet feeding promoted body weight gain, increased the fat pads and the adipose index, resulting in glucose intolerance and IR. This was accompanied by alterations of mitochondrial ultrastructure in the gastrocnemius muscle, reduction in superoxide dismutase (SOD) activity and PGC-1α mRNA expression, as well as elevation of protein carbonylation. These effects were partially reversed with physical activity
Chen et al., 2018 [13]	Male Sprague-Dawley rats fed HFD/streptozotocin (35 mg kg^−^1) for 5 weeks. Thereafter they were injected of puerarin at 100 mg kg^−1^, i.e.) for 4 weeks	HFD-feeding downregulated the expression (mRNA/protein) of PGC-1α, NRF1/2, and TFAM. This was correlated with enhanced levels of reactive oxygen species (ROS), and the oxidation of fatty acids in the muscle. Treatment with puerarin was able to ameliorate these effects
Kim et al., 2018 [34]	Male C57BL/6 mice fed HFD for 10 weeks. Thereafter, they were fed HFD with chicoric acid (at 0.03%, w/w) for 6 weeks	HFD-feeding caused glucose intolerance and IR, which was consistent with dysregulated mRNA expression of PGC-1α, NRF1, NRF2, and TFAM. These effects were ameliorated with diet rich in chicoric acid
Branco et al., 2019 [10]	Male C57BL/6 mice were subjected to a protein-restricted (6% protein-restricted diet) diet for 6 weeks before HFD or not for 8 weeks	Protein-restricted diet was associated with lower weight gain and fat accumulation and did not show an increase in fasting plasma glucose and insulin levels compared to mice on HFD only. Protein restriction also promoted skeletal muscle energy expenditure, as designated by increased citrate synthase, and PGC-1α protein content, and higher levels of malate and α-ketoglutarate
Falcão-Tebas et al., 2019 [18]	Male Sprague–Dawley rats fed HFD for 10 weeks before mating with chow-fed dams. Female offspring remained sedentary or performed moderate intensity treadmill exercise from 5 to 9 weeks of age	A paternal HFD-feeding negatively affected female rat offspring glucose tolerance, pancreatic morphology and insulin secretion and sensitivity. These effects occurred independent of mitochondrial H_2_O_2_ production and PGC-1α protein expression. Exercise intervention did not affect mitochondrial respiration, however improved that of offspring sired by HFD fed fathers in adulthood
Pattanakuhar et al., 2019 [63]	Female Wistar rats fed HFD for 27 weeks. These rats were further subjected to caloric restriction and endurance exercise 5 times per week for 7 weeks	HFD-feeding promoted IR and impaired mitochondrial function. This was associated with, enhanced ROS and membrane depolarization. Caloric restriction and exercise reversed these effects, leading to enhanced protein expression of PGC-1α, and improved mitochondrial biogenesis
Zheng et al., 2020 [85]	Male C57BL/6J mice fed HFD for 12 weeks and injected with streptozotocin (STZ) for 7 days. These mice were further subjected to treadmill exercise for 8 weeks	HFD with STZ increased body weight and impaired glucose/insulin tolerance but this was improved by 8 weeks of highly intensive exercise. Interestingly, sedentary mice exposed to HFD with STZ displayed reduced protein expression of PGC-1α albeit, but highly intensive exercise showed an improvement in the expression of PGC-1α as well as that of mitochondrial fusion proteins

Notably, combining caloric restriction and endurance exercise (five times per week for 7 weeks) could improve mitochondrial biogenesis in skeletal muscles of Wistar rats fed with HFD for 27 weeks [63]. These effects were consistent with modulation of PGC-1α expression, amelioration of insulin resistance, reduction in toxic levels of ROS and improvement in mitochondrial dynamics and skeletal muscle function. These effects could also be observed independent of caloric restriction in rodents subjected to regular physical exercise, either treadmill exercise or swimming for approximately 8 weeks [6, 85]. Where it was shown that regular physical exercise could stimulate PGC-1α expression within the skeletal muscle to enhance insulin sensitivity, improve mitochondrial ultrastructure, and increase intracellular antioxidant response [6, 85]. The positive effects of caloric restriction and regular physical exercise on improving skeletal muscle function are widely acknowledged [38, 51]. Evidence regarding the influence of physical exercise on maternal diet-induced metabolic dysregulations that involve PGC-1α regulation is very limited and remains inconclusive [18].

Antioxidants and natural products rich in these active ingredients are increasingly investigated for their role in alleviating HFD-induced skeletal muscle alterations in preclinical models [32, 62]. Here, injection with the isoflavone puerarin, at 100 mg/kg for 4 weeks, could downregulate the expression of a range of genes involved in mitochondrial biogenesis and oxidative phosphorylation, such as PGC-1α, NRF1/2, and transcription factor A (TFAM) in HFD-fed Wistar rats [13], whereas treatment with HFD containing the phenylpropanoid chicoric acid, at 0.03%, w/w for 6 weeks, was associated with improved glucose and insulin metabolism, while also reversing mitochondrial biogenesis and oxidative phosphorylation within the skeletal muscle in C57BL/6 mice fed HFD for 10 weeks [34]. Collaboratively, our group has increasingly reported on the potential therapeutic effects of bioactive compounds with abundant antioxidant effects, including polyphenols which are highly present in fruits and vegetables, in improving skeletal muscle function by ameliorating insulin resistance and targeting improving mitochondrial function [58, 59].

## Summary and concluding remarks

Animal models have undoubtedly become fundamental in providing a platform to uncover pathological mechanisms that may be involved during the early development or progression of this condition [19]. This review confirms that partial exposure to HFD feeding is associated with impairments in mitochondrial respiration and initiating of insulin resistance. Apparently, these effects (during early development of disease) can cause insulin resistance and mitochondrial dysfunction by obstructing skeletal muscle adaptations in part by reducing the activity of PGC-1α and insulin signaling pathway. Notably, other PGC-1α-related transcriptional factors like TFAM and NRF1 were also suppressed during this process. Interestingly, it has already been proposed that stimulation of PGC-1α, together with associated factors like AMPK, SIRT1, and PPARγ are necessary for the skeletal muscle to handle FFA overload and improve insulin signaling [37, 50]. These results further indicate that long-term exposure to lipid overload (likely during late development of disease) might severely affect the mitochondrial oxidative capacity, causing protein loss or muscle wasting, as previously reported [1, 4, 57], further indicating the importance of targeting PGC-1α in improving skeletal muscle adaptations through stimulating mitochondrial biogenesis and enhancing insulin sensitivity under toxic conditions of lipid overload. Importantly, summary of findings within this review are in line with research that has been published over the years indicating the central role of PGC-1α during the development of insulin resistance and mitochondrial dysfunction within the skeletal muscle in experimental models of HFD [9, 11, 77]. In fact, others have showed that overexpression of this transcriptional factor within the skeletal muscle is sufficient to improve insulin sensitivity in rats [8]. However, more cellular mechanisms are controlled by PGC-1α and should be explored to better understand this paradigm in which HFD feeding provokes a disconnect between mitochondrial function and insulin signaling, through the dysregulations in PGC-1α within the skeletal muscle. Also, beyond the use of physical exercise [61, 78], large scale studies are required to test whether pharmacological stimulation or stimulation of this transcriptional factor can be beneficial in reversing pathological consequences of the metabolic disease.

## Data Availability

Data related to search strategy, study selection, and extraction items will be made available upon request after the manuscript is published.
